# Prevalence and risk factors associated with *Ehrlichia* infections in smallholder dairy cattle in Nairobi City County, Kenya

**DOI:** 10.14202/vetworld.2019.1599-1607

**Published:** 2019-10-24

**Authors:** Shepelo Getrude Peter, Daniel Waweru Gakuya, Ndichu Maingi, Charles Matiku Mulei

**Affiliations:** 1 Department of Clinical Studies, Faculty of Veterinary Medicine, University of Nairobi, Kenya; 2 Department of Veterinary Pathology, Microbiology and Parasitology, Faculty of Veterinary Medicine, University of Nairobi, Kenya

**Keywords:** *Ehrlichia ruminantium*, enzyme-linked immunosorbent assay, inclusion bodies, microscopy, tick-borne disease

## Abstract

**Background and Aim:**

Ehrlichiosis caused by *Ehrlichia ruminantium* is a tick-borne disease of great economic importance in cattle production worldwide. Despite its economic impact, limited knowledge is available on its epidemiology in Africa, including Kenya. Suspected cases of *E. ruminantium* infections have been reported in the recent past to the University of Nairobi’s Veterinary Hospital, prompting the need to investigate their possible re-emergence. Therefore, this study was aimed at determining the prevalence of *E. ruminantium* among smallholder dairy cattle in Nairobi City County and to assess potential risk factors. This knowledge may guide the development of appropriate control strategies of ehrlichiosis, subsequently reducing associated losses.

**Materials and Methods:**

A total of 107 smallholder dairy farms from Nairobi City County were recruited for the study. Blood samples were collected from 314 apparently healthy dairy cattle, and Giemsa-stained blood smears were screened under the microscope for *Ehrlichia* species. A commercial antigen enzyme-linked immunosorbent assay (ELISA) kit was then used to confirm the presence of the infections in serum samples. A pre-tested questionnaire was used to collect data on management practices that may be potential risk factors. A univariate and mixed-effects logistic regression was then used to determine significant risk factors.

**Results:**

On microscopy, 79.3% (249/314) of the sampled animals had *Ehrlichia*-like inclusion bodies in white blood cells, though only 18.6% (95% confidence interval [CI] 14.2-23.0) of these were confirmed to be *E. ruminantium* on ELISA. A farm-level prevalence of 35.5% (95% CI 27.0-45.3) was reported. Female-headed households (p=0.013), farms in Langata region (p=0.027), cleaning of cowsheds fortnightly (p=0.019), and roofing of cowshed (p=0.022) were factors significantly associated with *E. ruminantium* infections.

**Conclusion:**

There is a relatively high prevalence of *E. ruminantium* infections in apparently healthy cattle in smallholder dairy farms in this area, warranting control measures. It is critical to improve animal welfare-related factors, such as cowshed cleaning and roofing, as well as the strategic location of farms, especially, since reservoirs may reduce infection levels in the farms, in relation to wildlife. However, since *Ehrlichia*-like inclusion bodies other than those of *E. ruminantium* were observed in this study, there is a need to investigate further these factors and the possibility of other *Ehrlichia* species infecting cattle in the study area.

## Introduction

Ehrlichiosis is a broad term referring to tick-borne diseases (TBDs) of multiple animal species caused by obligate intracellular organisms of the white blood cells [1]. *Ehrlichia ruminantium* is the main *Ehrlichia* species infecting cattle in Africa and the Caribbean Islands [2]. It is transmitted by infected ticks in the genus *Amblyomma* [2,3]. Despite *E. ruminantium* being among the most important TBD affecting millions of cattle in sub-Saharan Africa and being an OIE listed disease [4-7], there is limited information on its epidemiology in Africa, including Kenya [8]. Instead, other TBDs, such as East Coast Fever, anaplasmosis, and babesiosis, have been considered the most important for cattle throughout Eastern Africa, including Kenya [9,10]. This may be a consequence of certain reports of the low prevalence of *E. ruminantium* in some parts of Africa and even Kenya, including 0.6% in Ethiopia [11], 0.4% in Kenya [12], and 1.1% and 1.7% in Nigeria and Uganda, respectively [13,14]. Due to the high demand for milk, market forces and the convenience of urban areas, there has been a constant rise in dairy cattle keeping in urban areas of Kenya [15]. These smallholder dairy production systems in urban and peri-urban areas experience unique challenges where their substandard animal husbandry practices compromise animal welfare, thereby predisposing the cattle to stressful conditions and, as a result, various diseases [16].

TBDs are the most important diseases affecting these smallholder dairy farms, mainly due to dynamic tick vectors, increasing the susceptibility of the exotic breeds kept in the farms [17]. *E. ruminantium* infections cause severe economic losses in Africa, where approximately 150 million animals are at risk of infection [7], thereby negatively affecting livelihoods that depend on cattle. The estimation of these losses in endemic areas is complicated by the fact that farmers do not provide regular reports, definitive diagnoses are hardly delivered [7], and infections often coincide with other TBDs such as anaplasmosis and East Coast Fever [2]. Despite this, the few economic studies that have been carried out have pointed to quite substantial economic losses. In Zimbabwe, losses of USD 5.6 million/year have been reported, with acaricide costs accounting for 76% and milk losses accounting for 18% of the total cost [18]. In a 3-year follow-up study in Ethiopia, losses from mortalities, cost of acaricides and antibiotics, and losses in milk and meat have resulted in total costs of USD 7884.67 million [8]. In Tanzania, economic losses of USD 22.6 million/year have been recorded, with cattle mortality accounting for 8.8 million USD [19].

Suspected cases of ehrlichiosis have been reported since 2014 at the University of Nairobi Veterinary Hospital, which mainly serves Nairobi City County and its peri-urban areas. These cases present with non-specific clinical signs such as inappetence, unthriftiness and, at times, death. When they are screened by a blood smear microscopy, *Ehrlichia*-like inclusion bodies have been shown in the white blood cells of affected animals. The difficulty in diagnosing *E. ruminantium* infections due to their non-pathognomonic clinical signs has been emphasized by Allsopp and has resulted in the underestimation of economic losses, especially in endemic areas including Kenya [2].

This study was therefore aimed at determining whether the inclusion bodies observed in blood samples collected from smallholder dairy farms within Nairobi City County were those of *E. ruminantium*, and to assess potential risk factors, therefore warranting the development of appropriate control measures in these farms.

## Materials and Methods

### Ethical approval

The study was approved by Biosecurity, Animal Use and Ethics Committee, Faculty of Veterinary Medicine, University of Nairobi (FVM BAUEC/2016/122). Experiments were carried out in accordance with the guidelines laid down by the International Animal Ethics Committee or Institutional Ethics Committee and in accordance with local laws and regulations.

### Informed consent

The study was discussed with owners of the animals who were to be recruited and thereafter they signed written consent. They were free to make the decision to join the study or withdraw.

### Study area and study population

The study was conducted in Nairobi City County’s Nairobi City, which is the capital city of Kenya and is located at 1.28333 latitude and 36.81667 longitude and 1795 m above sea level. The county has 17 sub-counties, the highest number of administrative units in a single county in Kenya. The city has a population of slightly over 3 million people. In the peri-urban areas of the city, there are the smallholder dairy production systems established to meet the high demand for the milk of the city. The previously reported incidence of TBD in these peri-urban areas of Nairobi was 7.8% [10].

### Study design

**Figure-1:**
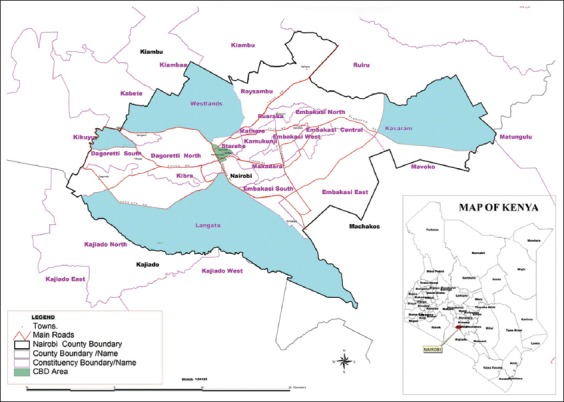
Map of Nairobi City County highlighting the sub-counties that were sampled. [Source: Independent Electoral and Boundaries Commission, Kenya https://www.iebc.or.ke/uploads/resources/WHXao7x83D.pdf]

Nairobi City County was divided into four quadrants centered around the city’s central business district (Figure-1). In each of the quadrants, purposive sampling was used to identify the sub-county with the highest cattle population, which was included in the study. The sub-counties identified in each quadrant were Dagoretti, Langata, Ruai, and Westlands. Transect walks were then used to identify smallholder dairy farms in the sub-counties. Smallholder dairy farms were described as those with between 2 and 10 dairy cattle [20]. In each transect the tenth; dairy unit was included in the study depending on the owner’s willingness to participate. Otherwise, if they refused to give consent, the next dairy unit was recruited. Animals were grouped into age groups (calves <12 months, yearlings 12 - ≤24 months, and adults >24 months). A maximum of two animals in each age group was included in the study. Where there were more than two animals in a specified age group, two were randomly selected. A pre-tested questionnaire was administered to the principal farmer or stockman who spent the most time with the cattle to collect farm-level management factors that were thought to be associated with *Ehrlichia* infections. Using an expected prevalence of *E. ruminantium* of 4.5% [21] and sample size calculation formulae according to Charan and Biswas [22], the minimum target sample size was found to be 66 cattle.

### Data and sample collection

Data on the various farm-level factors, especially management practices (feeding system, source of fodder, cowshed cleaning, tick control, nature of housing, and introduction of new animal) and animal level factors (age, sex, breed, and lactation status) were collected using the pre-tested questionnaire. Blood was collected from the coccygeal vein and put in vacutainers with clot activator and allowed to stand for 2 h. The serum was then decanted in a labeled Eppendorf tube and centrifuged for 10 min at 3000× *g*, then decanted into the final Eppendorf tube and stored at −20°C until further analysis. A blood smear was immediately prepared at the farm, fixed using absolute ethanol, and later Giemsa-stained.

### Laboratory analysis

Microscopy was done using a light microscope at 1000× oil emersion on the Giemsa-stained smears where the white blood cells (neutrophils, lymphocytes, and monocytes) were assessed for any cytoplasmic inclusion bodies. Antigen enzyme-linked immunosorbent assay (ELISA) for *E. ruminantium* was done on the harvested serum using commercial kits BIOS microwell ELISA diagnostic systems, USA, following the manufacturer’s instructions.

### Statistical analysis

Questionnaire data were input into Excel 2016 before it was exported to Stata 15.0 for analysis. Descriptive statistics expressed as proportions were computed for the farm and animal level factors in the study area. Univariate logistic regression was used to test for associations between the various possible risk factors as explanatory variables and *E. ruminantium*-positive outcome on ELISA. Factors with p≤10% were analyzed using mixed-effects logistic regression model followed by step-wise elimination such that only factors with p<5% were left in the final model and were identified as being statistically associated with *E. ruminantium* infections.

## Results

### Microscopic examination

A total of 314 blood samples were collected from cattle in 107 smallholder dairy farms from the four selected regions in Nairobi City County. Inclusion bodies were observed in white blood cells in 79.3% (249/314) (95% confidence interval [CI] 74.4-83.6) of the samples examined. The Dagoretti region had the highest number of animals, with inclusion bodies in their white blood cells, totaling 89.5 (94/105). The Westlands and Ruai regions reported the lowest numbers of animals with inclusion bodies among the animals examined (Table-1).

**Table 1:** Distribution of number and percentage of *Ehrlichia-* like inclusion bodies in four different regions in Nairobi City County, Kenya.

Region	Number of samples	*Ehrlichia*-like inclusion bodies present (%)
Dagoretti region	105	94 (89.5)
Langata region	60	50 (83.3)
Ruai region	102	72 (70.6)
Westlands region	47	33 (70.2)
Total	314	249 (100)

**Figure-2:**
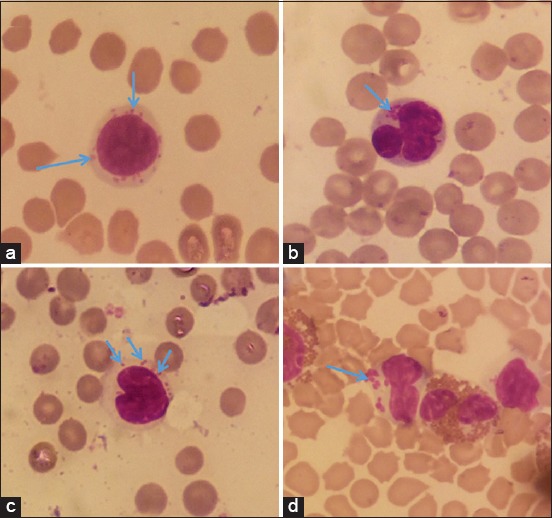
*Ehrlichia-like* inclusion bodies (blue arrows) as observed under a light microscope (1000×) oil immersion in a lymphocyte (a), neutrophil (b), and a monocyte (c and d).

Out of the 249 samples that were positive on microscopic analysis, 145 samples that had clean blood smears were scrutinized to assess the specific white blood cells that had *Ehrlichia*-like inclusion bodies. Of all the cells that were examined, 49.7% (72/145) had *Ehrlichia*-like inclusion bodies in monocytic cells, while only 4.8% (7/145) were in granulocytic cells. The other 45.5% (66/145) of the samples had inclusion bodies in both granulocytic and monocytic cells. Among the monocytic cells, the lymphocytes were most commonly affected, representing 37.2% of the cells (54/145) followed by monocytes, representing 12.4% (18/145) of the cells. Figure-2 shows the *Ehrlichia-* like inclusion bodies as they appear in different white blood cells.

### Determination of *E. ruminantium* infections in smallholder dairy farms in Nairobi City County using ELISA

Antigen ELISAs were carried to confirm whether the *Ehrlichia*-like inclusion bodies were those of *E. ruminantium* or not. The ELISA was done on 296 of the samples that were examined by microscopy, of which 18.6% (55/296) (95% CI [14.2-23.0]) were positive for *E. ruminantium*. Of all the sampled farms, nearly a third of them, representing 35.5% (38/107) (95% CI [27.0-45.3]), had animals positive for *E. ruminantium*.

### Description of factors associated with *E. ruminantium* infections in smallholder dairy farms in Nairobi City County

Table-2 describes the distribution of the various factors among the study’s farms. *Ehrlichia* positive farms were more prevalent in female-headed households, representing 60% (23/38), than in male-headed households, representing 39.5% (15/38) of the farms. The majority of the positive farms were in the Ruai region 42.1% (16/38), although fewer farms were sampled from the Westlands and Langata Regions. There were more farms with *E. ruminantium* infections in which employees had only attained a primary education or lower, representing 44.8% (17/38) of the farms, in comparison to farms in which employees had attained a higher educational level, representing 15.8% (6/38) of the farms. There were more *E. ruminantium* infections, representing 57.8% (22/38), in farms where the owners had additional sources of income from businesses, compared to farms in which the owners depended solely on livestock or their salary, representing 23.7% (9/38) of farms.

**Table 2:** Description of farm and animal level factors associated with *Ehrlichia ruminantium* infections among 296 dairy cattle in 107 smallholder dairy farms across Nairobi City County.

Parameter	Description	*Ehrlichia ruminantium* positive (%)
Farm-level factors		
Gender of household head	Female-headed household (n=42)	23 (60.0)
	Male-headed household (n=65)	15 (39.5)
Region	Dagoretti region (n=52)	12 (31.6)
	Ruai region (n=39)	16 (42.1)
	Westlands region (n=7)	4 (10.5)
	Langata region (n=9)	6 (15.8)
Employee present	Yes (n=67)	24 (63.2)
	No (n=38)	14 (36.8)
Employee’s education level	Employees’ highest level of education was primary (n=42)	17 (44.8)
	Employees’ highest level of education was secondary (n=23)	6 (15.8)
	Employees’ highest level of education was tertiary (n=4)	1 (2.6)
Farmer’s education level	Owners’ highest level of education was primary (n=27)	11 (28.9)
	Owners’ highest level of education was secondary (n=42)	12 (31.6)
	Owners’ highest level of education was tertiary (n=38)	15 (39.5)
Farming system	Farming system involved livestock only (n=7)	6 (15.8)
	Farming system involved livestock and crops (n=100)	32 (84.2)
Other sources of income	No other source of income (n=34)	9 (23.6)
	Additional income from salary (n=20)	7 (18.4)
	Additional income from business (n=53)	22 (57.8)
Feeding system	Stall feeding only (n=86)	29 (76.3)
	Free grazing only (n=3)	2 (5.3)
	Stall feeding and free grazing (n=18)	7 (18.4)
Source of fodder	Fodder sourced from own farm (n=38)	10 (26.3)
	Fodder sourced from own farm and purchase (n=32)	15 (39.5)
	Fodder is purchased and cut from roadside grazing (n=37)	13 (34.2)
Hay feeding	Fed on hay (n=90)	33 (86.8)
	Not fed on hay (n=17)	5 (13.2)
Fencing of pastureland	Pastureland fenced (n=89)	32 (84.2)
	Pastureland not fenced (n=18)	6 (15.8)
Cleaning of cowshed	Cowshed cleaned daily (n=78)	23 (60.5)
	Cowshed cleaned every other day (n=10)	3 (7.9)
	Cowshed cleaned fortnightly (n=19)	12 (31.6)
Type of cowshed floor	Earthen cowshed floor (n=19)	10 (26.3)
	Cemented cowshed floor (n=60)	19 (50.0)
	Cowshed floor has stones (n=28)	9 (23.7)
Presence of cowshed bedding	Bedding present on cowshed floor (n=52)	18 (47.4)
	Bedding absence on cowshed floor (n=55)	20 (52.6)
Presence of cowshed roof	Roof present on cowshed (n=100)	34 (89.5)
	Roof absent on cowshed (n=7)	4 (10.5)
Tick control on cattle	Practicing tick control (n=79)	29 (76.3)
	Not practicing tick control (n=28)	9 (23.7)
Frequency of tick control	Acaricide applied weekly (n=47)	17 (44.7)
	Acaricide applied monthly (n=40)	19 (50.0)
	Acaricide applied every 3 months (n=20)	2 (5.3)
Method of acaricide application	Hand spray (n=96)	31 (81.6)
	Pour on (n=8)	5 (13.2)
	Other methods (n=3)	2 (5.3)
Presence of new animal in the herd	Introduction of new animals (n=31)	12 (31.2)
	Farms with no new animals introduced (n=76)	26 (68.4)
Animal-level Factors		
Age of the animal	Calves (n=80)	13 (4.4)
	Yearlings (n=73)	12 (4.1)
	Adults (n=143)	30 (10.1)
Animal breed	Friesian (n=183)	27 (9.1)
	Guernsey (n=10)	2 (0.7)
	Ayrshire (n=55)	4 (1.4)
	Indigenous (n=39)	22 (7.4)
	Jersey (n=9)	0 (0)
Sex of the animal	Female (n=268)	46 (15.5)
	Male (n=28)	9 (3.0)
Lactational status	Lactating (n=122)	22 (74.3)
	Pregnant (n=11)	3 (1.0)
	Calves/heifers/male (n=163)	30 (10.1)

Stall feeding was the main method of feeding the dairy cattle, and the majority of the infected farms, representing 76.3% (29/39), were in this category. *E. ruminantium* infections were lower in farms in which fodder fed to the animals was sourced from their own farm, representing 26.3% (10/38) of farms, in comparison to farms in which the fodder was purchased. In addition, farms that supplemented the fodder with hay feeding had high infection rates, of 86.8% (33/38), compared to those that did not feed hay 13.2% (5/38). Infection rates were also high in farms in which the cowshed was cleaned on a daily basis, representing 60.5% (23/38) of farms, and the cowshed floor was cemented, representing 50% (19/38) of the farms. Tick control was a major practice in the majority of the study farms, representing 73.8% (79/107) of the farms, though *E. ruminantium* infections remained high, affecting 76.3% (29/38) of farms, especially in farms implementing hand spraying 81.6% (31/38).

The majority of animals sampled were adults, representing 48.3% (143/296), and accounted for the highest age group with *E. ruminantium* infections, representing 10.1% (30/296). The main breed kept in the study farms was Friesian 61.8% (183/296), and, being dairy farms, the majority of animals were females 90.5% (268/296).

### Analysis of risk factors associated with *E. ruminantium* infections among 296 dairy cattle across 107 smallholder dairy farms in Nairobi City County

On univariate analysis, factors found to be statistically significant, at p≤0.1, were the gender of household head, region, farming systems, cleaning of cowshed, type of cowshed floor, presence of cowshed roof, and breed of the animal (Table-3). A mixed-effects logistic regression analysis revealed that the factors significantly associated with seropositivity were farms in which the household head was female (p=0.013), farms from the Langata region (p=0.027), cleaning of the cowshed every fortnight (p=0.019), and farms in which the cowsheds had roofs (p=0.022) (Table-4). Farms in which the household head was female were 2.4 times more likely to have *E. ruminantium* infections than male-headed households. Farms in the Langata Regions were 2.9 times more likely to be infected with *E. ruminantium* than farms in the Dagoretti region, while farms that cleaned their cowsheds fortnightly were 3.6 times more likely to be infected with *E. ruminantium* that those that cleaned their cowsheds on a daily basis. Farms in which cowsheds had no roofs were 4.7 times more likely to be infected by *E. ruminantium* than those which had cowshed roofs.

**Table 3:** Univariate logistic regression of factors associated with *Ehrlichia ruminantium* among 296 dairy cattle across 107 smallholder farms in Nairobi City County.

Parameter	Estimate	p-value	95% Confidence interval	

			Lower	Upper
Gender of household head	−1.356	0.003*	−2.194	−0.514
Region	0.263	0.099*	−0.049	0.5745
Gender of owner	0.117	0.671	0.655	0.421
Employee present	−0.336	0.471	−1.250	0.578
Farmer’s education level	−0.204	0.454	−0.738	0.330
Farming system	−0.204	0.005*	−2.948	−0.526
Other sources of income	0.067	0.795	−0.438	0.572
Duration of farming	0.016	0.995	−0.031	0.031
Feeding system	−0.019	0.950	−0.571	0.536
Source of fodder	0.266	0.901	−0.553	0.488
Feeding of hay	−0.057	0.929	−1.314	1.200
Area for fodder growing	−0.133	0.258	−0.363	0.097
Fencing of pastureland	−0.158	0.773	−1.230	0.915
Cleaning of cowshed	0.989	<0.001*	0.514	1.464
Type of cowshed floor	−0.839	0.006*	−1.437	−0.241
Presence of cowshed bedding	−0.371	0.389	−1.215	0.473
Presence of cowshed roof	−2.551	<0.001*	−3.720	−1.382
Tick control on cattle	0.309	0.559	−0.727	1.344
Frequency of tick control	−0.108	0.781	−0.866	0.651
Method of acaricide application	0.643	0.279	−0.521	1.807
New animals in the herd	0.008	0.986	−0.919	0.936
Age of the animal	0.171	0.423	−0.248	0.590
Breed of the animal	0.263	0.062*	−0.013	0.540
Sex of the animal	−0.668	0.212	−1.717	0.380
Lactation status	−0.023	0.898	−0.380	0.333
Parity	−0.064	0.491	−0.248	0.119

* Factors statistically significant at p≤0.1

**Table 4:** Mixed effects logistic regression analysis of the factors significantly associated with *Ehrlichia ruminantium* among 296 dairy cattle across 107 smallholder farms in Nairobi City County.

Parameter	OR	95% Confidence interval		p-value

		Lower	Upper	
Female household head	2.4	1.2	5.3	0.013
Male household head	1			
Ruai region	1.0	0.4	2.6	0.950
Westlands region	2.2	0.6	8.2	0.221
Langata region	2.9	1.1	7.6	*0.027
Dagoretti region	1			
Every other day cleaning of cow shed	2.5	0.6	11.2	0.216
Cleaning of cow shed fortnightly	3.6	1.2	10.7	*0.019
Daily cleaning of cow shed	1			
Cowshed without a roof	4.7	1.2	17.4	*0.022
Cowshed with a roof	1			

* Factors statistically significant at p≤0.05. OR=Odds ratio

## Discussion

In this study, *Ehrlichia*-like inclusion bodies were observed in the cytoplasm of various white blood cells. Although microscopic examination of endothelial cells from the brain is the common method of diagnosing *E. ruminantium*, the inclusion bodies of the parasite can still be observed in the neutrophils on stained blood smears [1,23]. In this study, inclusion bodies were also observed in neutrophils as well as in other white cells such as lymphocytes and monocytes. Some of the samples that revealed inclusion bodies on microscopy were negative on ELISA, possibly indicating the presence of other species of *Ehrlichia* in the cattle since they all parasitize white blood cells [24]. Despite microscopy being cheap and easily available, the low sensitivity and occasional misdiagnosis due to the presence of artifacts in the smears greatly limit its use [25]. These limitations may explain the high number of *Ehrlichia*-like inclusion bodies observed in this study.

The current study used antigen ELISA to detect *E. ruminantium* in an attempt to overcome the limitations linked to cross-reactivity with other *Ehrlichia* species [26]. *E. ruminantium* was detected in apparently healthy cattle, an observation similar to that of Matos *et al*. [27] but inconsistent with Kelly *et al*. [28], who points out that *E. ruminantium* always presents with clinical disease. This may suggest that non-pathogenic strains of the parasite may be in circulation, or those carrier animals have the parasites, thereby posing a threat to susceptible animals [2].

The overall prevalence of *E. ruminantium* in cattle reported in this study was 18.6%, similar to the 15% reported in Mozambique [27], though lower than 50% reported by Swai *et al*. [29] in Tanzania and 33% in Zimbabwe [26] and higher than 4.5% in Uganda [21] and 4.1% in Ethiopia [25]. This wide variability may suggest differences in factors related to cattle management across various areas. The prevalence recorded in the present study is sufficiently high; however, to warrant the implementation of appropriate control strategies since there may be a risk of clinical disease if susceptible animals are present [29]. The herd prevalence was 35.5%, at nearly twice the animal prevalence, possibly indicating that *E. ruminantium* infection in this area represents a herd health concern rather than an individual animal’s problem [29].

Management practices such as acaricide application and frequency of dipping had previously been reported to be risk factors for *E. ruminantium* [29,30]. However, these factors were not found to be significant risk factors in the current study. Despite the farmers using acaricide for tick control, therefore ehrlichiosis, its mishandled such as through wrong dilution and application procedures may have led to possible development of ticks’ resistance [2,5]. In addition, most of the farms outsourced hay to feed their dairy cattle mainly because they own small land sizes, therefore inadequate fodder for the animals, a common feature of smallholder dairy production units in urban and peri-urban areas in Kenya [16]. The ticks can be carried from the grasses to the stall-fed animals, leading to possible infections.

Farms that cleaned the cowshed fortnightly had increased odds of *E. ruminantium* infections than those that cleaned them daily. Infrequent removal of slurry from cowsheds has been shown to compromise animal welfare in smallholder dairy units, especially because the housing designs are usually poor, thereby forcing the animals to lie on the slurry for long hours at a time [16]. Poor animal welfare, on the other hand, increases the stress of the affected animals, greatly compromising their immunity and predisposing them to other diseases [31]. This may explain the high number of infected animals in this study. Roofing is part of animal housing that provides protection from extreme weather [32]. Therefore, without roofs in the cowsheds, stressors such as excess cold or heat compromise animals’ immunity, possibly explaining the increased rate of *E. ruminantium* infections in farms with cowsheds without roofs [33].

The farms from the Langata region were more likely to have animals infected with *E. ruminantium* compared to other regions, possibly because this region borders Nairobi National Park and its wild animals, such as the African buffalo, black wildebeest, and eland, since they have been known to be reservoirs of ehrlichiosis, are associated with increased risk of infection [34].

Farms in which the household head is female were more likely to have animals infected with *E. ruminantium* than those in which the household head is male. These findings are similar to those of Sungirai *et al*. [5] who noted that women are less educated in the management of dairy enterprises, thereby increasing their risk of disease. Moreover, Tola *et al*. [35] also noted that women undertake the majority of the labor in dairy enterprises, minimizing their time for decision making, such as with regards to disease management.

A limitation of the current study was that sub-counties were not randomly selected since this is an urban-set up; therefore, dairy farms were only concentrated in certain sub-counties. However, the farms included in the study were well representative of Kenyan urban smallholder dairy farms; keeping 2-10 animals and practicing sub-optimal animal husbandry as described by Nguhiu-Mwangi *et al*. [16].

## Conclusion

There is a relatively high prevalence of *E. ruminantium*, both at the animal level (18.6%) and at the herd-level (35.5%), in smallholder dairy production systems in the study region, warranting control measures to be put in place. Farm-level factors that predispose to *E. ruminantium* infections were identified as infrequent cowshed cleaning, lack of roofing, regions in close proximity to wildlife and female household heads. Moreover, there is a need to further characterize the current strains of *E. ruminantium* in circulation among dairy cattle in this area, especially to determine their virulence since, as reported elsewhere; some may have the zoonotic potential [36]. Increasing the frequency of cowshed cleaning to remove the slurry and roofing cowsheds will greatly improve animal welfare, subsequently boosting their immunity and controlling *E. ruminantium* infections.

## Authors’ Contributions

SGP collected, analyzed the samples, and wrote the manuscript. DWG collected samples and interpreted the data. NM and CMM analyzed and interpreted the data. All the authors read and approved the final manuscript.
